# Severe Dengue Epidemics in Sri Lanka, 2003–2006 

**DOI:** 10.3201/eid1502.080926

**Published:** 2009-02

**Authors:** Nalaka Kanakaratne, Wahala M.P.B. Wahala, William B. Messer, Hasitha A. Tissera, Aruna Shahani, Nihal Abeysinghe, Aravinda M. de Silva, Maya Gunasekera

**Affiliations:** Genetech Research Institute, Colombo, Sri Lanka (N. Kanakaratne, M. Gunasekera); University of North Carolina School of Medicine, Chapel Hill, North Carolina, USA (W.M.P.B. Wahala, W.B. Messer, A.M de Silva); Ministry of Health, Colombo (H.A. Tissera, N. Abeysinghe); Apollo Hospital, Colombo (A. Shahani); 1 Deceased.

**Keywords:** Dengue, dengue hemorrhagic fever, dengue serotype 3, flavivirus, molecular epidemiology, phylogeny, emerging virus, Sri Lanka, Indian subcontinent, research

## Abstract

Recent emergence of dengue hemorrhagic fever in the Indian subcontinent has been well documented in Sri Lanka. We compare recent (2003–2006) and past (1980–1997) dengue surveillance data for Sri Lanka. The 4 dengue virus (DENV) serotypes have been cocirculating in Sri Lanka for >30 years. Over this period, a new genotype of DENV-1 has replaced an old genotype. Moreover, new clades of DENV-3 genotype III viruses have replaced older clades. Emergence of new clades of DENV-3 in 1989 and 2000 coincided with abrupt increases in the number of reported dengue cases, implicating this serotype in severe epidemics. In 1980–1997, most reported dengue cases were in children. Recent epidemics have been characterized by many cases in children and adults. Changes in local transmission dynamics and genetic changes in DENV-3 are likely increasing emergence of severe dengue epidemics in Sri Lanka.

## Abstract 2

One-sentence summary for table of contents: Changes in transmission dynamics and virus genes are likely increasing emergence of severe epidemics in this country.

Dengue viruses (DENVs) are mosquito-borne flaviviruses that each year infect millions of persons living in tropical and subtropical regions of the world. Several hundred thousand of these infections, especially in children, progress to a life-threatening disease known as dengue hemorrhagic fever (DHF). Dengue has emerged in many regions of the world and the number of cases and the range of the virus continue to increase every year (*1*).

The DENV complex consists of 4 distinct serotypes, designated DENV-1, DENV-2, DENV-3, and DENV-4. Infection with 1 DENV serotype is believed to provide long-term immunity to the homologous serotype but not to the other serotypes (*2*). Thus, persons can be infected with multiple serotypes during their lifetime. People with a repeat (secondary) DENV infection have a greater risk for DHF than persons infected for the first time, indicating that preexisting serotype cross-reactive immunity is a risk factor for severe disease (*3*,*4*). Furthermore, all 4 serotypes of DENV can cause DHF, but within each serotype some genotypes or clades within genotypes are linked to severe disease and others to mild disease (*5*–*7*).

Factors driving global emergence of dengue fever (DF) and DHF are complex and include viral and host factors as well as environmental changes that favor transmission. The epidemiology of dengue in Sri Lanka is particularly interesting because before 1989 all 4 serotypes were present and many repeat infections occurred, but few cases of DHF were documented (*8*,*9*). Incidence of DHF dramatically increased in 1989, and hundreds to thousands of cases of DHF have been documented every year since (*8*). Genetic studies with DENV-3 strains from Sri Lanka demonstrated that viruses isolated before and after emergence of DHF belonged to 2 distinct clades (DENV-3, genotype IIIA and IIIB, respectively), indicating that DENV-3 strain differences are likely to have contributed to emergence of DHF (*7*).

The magnitude of DF and DHF epidemics in Sri Lanka has continued to increase; 2 of the largest epidemics occurred in 2002 and 2004. We report results from dengue surveillance and virologic studies conducted during 2003–2006 in Sri Lanka. We also compare recent (2003–2006) and past (1981–1997) surveillance data and virus isolates to better understand factors driving emergence of severe disease in Sri Lanka.

## Materials and Methods

### Sample Collection

Genetech Molecular Diagnostics Institute in Colombo, Sri Lanka, receives diagnostic specimens for dengue testing from clinics and hospitals in Colombo. This study used excess serum samples that remained after diagnostic testing. Only samples collected from patients with 1–4 days of fever were included in the study. All patient-identifying information was removed from specimens before their use in the study. The study was reviewed and approved by the Institutional Review Boards of the University of North Carolina, Chapel Hill, NC, USA, and the University of Sri Lanka, Peradeniya, Sri Lanka.

### National Dengue Data Collected by Ministry of Health, 1996–2005

DF and DHF are reportable diseases in Sri Lanka. All practicing doctors treating dengue patients are expected to report cases to local health officers, who report cases on a weekly basis to the Central Epidemiology Unit of the Ministry of Health in Colombo. National data reported in this article are based on cases reported to the Central Epidemiology Unit. A special investigation form for collection of detailed information is sent out by the Central Epidemiology Unit for each reported case of DF or DHF to the reporting health office and the treating hospital. Age-specific disease information in this article was compiled from these special investigation forms.

### Reverse Transcription–PCR for Detection and Serotyping of DENVs

Reverse transcription–PCR was performed by using the DV1 and DV3 primer set (*10*) and the ALD 1 and ALD 2 primer set (*11*) in 1 reaction. The DV primers amplify a 470-bp fragment of the nonstructural protein 3 (NS3) gene of all flaviviruses (*10*). The ALD1 and ALD2 primers amplify a 229–240-bp product from the 3′ untranslated region of all DENVs (*11*). The DV primers were not as sensitive as the ALD primers for detecting dengue infection. However, the 470-bp fragment amplified by the DV primers was used as the template in a second nested PCR to serotype the virus (*10*).

### Isolation of DENV

For virus isolation, 15 μL of serum was mixed with185 μL of minimal essential medium containing 2% fetal bovine serum and added to C6/36 cells growing in 6-well tissue culture plates. The inoculum was incubated for 1 hour at 28°C before adding 2 mL of medium and incubating for 10 days in a CO_2_ incubator at 28°C. Cells were tested for DENV by staining with monoclonal antibody 4G2, which binds to the envelope (E) protein of all 4 DENV serotypes. Supernatants were harvested from positive wells and frozen as P1 DENV stocks.

### Sequencing and Phylogenetic Analysis of DENV

The P1 stocks were used as a source of RNA for sequencing and genotyping viruses. Reverse transcription–PCR was performed with different primer pairs to amplify selected regions of the genome of DENV-1, -2, -3, or -4. For DENV-1, we amplified a 536-bp segment at the envelope-NS1 junction by using primers D1F 2034–2055 (5′-CCTTTTGGTGAGAGCTACATCG-3′) and D1R 2570–2551 (5′- ACACACACCCTCCTCCCATG-3′). For DENV-2, we amplified a 519-bp segment at the E-NS1 junction by using primers D2F 2050–2071 (5′-CCATTCGGAGACAGCTACATCA-3′) and D2R 2569–2548 (5′-GAGCCTTCTGGATAGCTGAAGC-3′). For DENV-3, we amplified a 1,057-bp segment encompassing part of the capsid (C) protein, the premembrane (preM) protein, and part of the E protein by using primers D3F 132–159 (5′- TCAATATGCTGAAACGCGTGAGAAACCG-3′) and D3R 1189–1171 (5′- CTCCTCAGGCAAAACCGCT-3′). For DENV-4, we amplified a 962-bp segment encompassing part of the C protein, preM protein, and part of the E protein by using primers D4F 137–162 (5′-TCAATATGCTGAAACGCGAGAGAACCG 3-′) and D4R 1099–1074 (5′-CCACTTCCTTGGCTGTTGTCTTGATC-3′).

Purified PCR products were sent to the University of North Carolina–Chapel Hill Genome Analysis Facility. Overlapping individual nucleic acid sequences were assembled by using VECTOR NTI (ContigExpress, Bethesda, MD, USA). Sequences were aligned and analyzed by using ClustalX (www.clustal.org), PAUP* (http://paup.csit.fsu.edu), PHYLIP (http://evolution.gs.washington.edu/phylip.html), and MEGA4 (www.megasoftware.net) software. All new virus sequences were deposited in GenBank (Appendix Table) for virus strains and sequences used to create the phylogenetic trees.

## Results

In January 2003, Genetech Molecular Diagnostics Institute in Sri Lanka began to test clinical specimens for DENV by PCR. Only samples collected from suspected dengue case-patients within the first 4 days of fever were tested. During 2003–2006, a total of 3,833 serum samples were received from hospitals and clinics. The number of samples tested ranged from 212 in 2003 to 1,686 in 2004 when Sri Lanka had a large DHF epidemic. Of the 3,833 samples, 930 (24%) were positive by PCR for DENV. On an annual basis, the proportion of positive samples was 39% in 2003, 22% in 2004, 18% in 2005, and 32% in 2006.

### Comparison of Dengue Data Collected at Genetech with Nationally Reported Data

**Figure 1:**
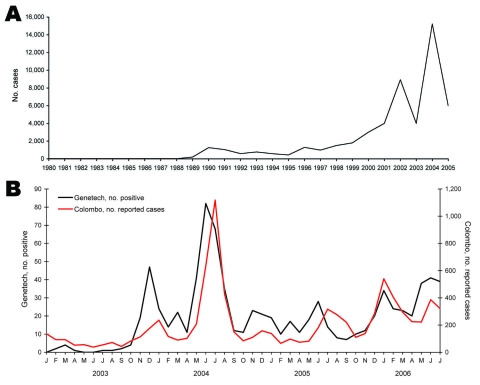
A) Dengue cases reported to the Epidemiology Unit, Ministry of Health, Sri Lanka (1981–2005). B) Comparison of monthly reported data for Colombo and Genetech for 2003–2006. Colombo data are based on cases reported to the Ministry of Health by hospitals and clinics within the Colombo Municipal Council. Genetech data are based on the number of PCR-positive cases detected each month.

Figure 1, panel A, shows dengue cases reported to the Ministry of Health in Sri Lanka during 1980–2005. Cases reported before 1996 are based on passive surveillance and outbreak investigations conducted by the Ministry of Health. DF and DHF were designated as reportable diseases in 1996, and data obtained since 1996 are based on mandatory reporting. Although DENVs were common in Sri Lanka and persons there were exposed to multiple infections, severe disease was rare before 1989 (*8*). The 1990s were characterized by small but regular epidemics of severe disease (Figure 1, panel A) (*8*). In the period since 2000, the magnitude of the epidemics has increased further; particularly large epidemics occurred in 2002 and 2004 (Figure 1, panel A).

Most of the dengue samples tested at Genetech were received from private hospitals and clinics in Colombo. Because dengue is a reportable disease in Sri Lanka, physicians are expected to report cases to the Ministry of Health. We compared monthly dengue data reported to the Ministry of Health from Colombo and data collected at Genetech during January 2003–April 2006. Data from Genetech closely mirrored cases reported to the Ministry of Health, indicating that the laboratory at Genetech can serve as a sentinel site for monitoring DENV activity in the Colombo region (Figure 1, panel B). The peak number of cases observed at Genetech preceded reported peaks by ≈1 month (Figure 1, panel B); these cases from 2 sources were significantly correlated (correlation coefficient 0.80).

### Circulating Dengue Serotypes

**Figure 2:**
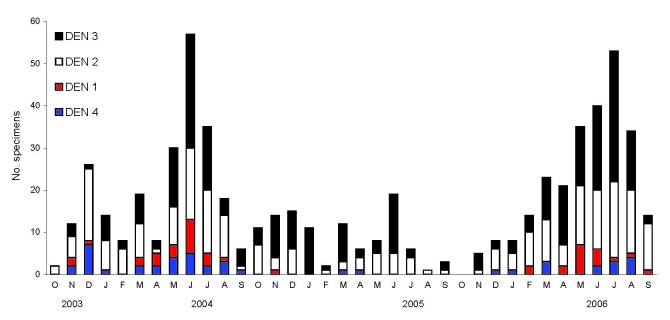
Relative abundance of dengue (DEN) virus serotypes in Sri Lanka. DEN-positive serum samples obtained from October 2003 through September 2006 were serotyped by reverse transcription–PCR.

Of 930 PCR-positive samples collected during 2003–2006, we serotyped 605 samples by nested PCR. DENV serotypes 2 (40%) and type 3 (46%) were common, and serotypes 1 (7%) and 4 (7%) were rare. We examined the relative abundance of each serotype at monthly intervals during October 2003–September 2006 (Figure 2). DENV-2 and DENV-3 were the dominant serotypes throughout the study period. DENV-1 and DENV-4 were also regularly isolated but in low numbers. All 4 serotypes were detected in 2004 and 2006. In 2005, DENV activity was low and DENV-1 was not identified in samples. These results demonstrate that although all 4 serotypes cocirculate in Sri Lanka, DENV serotypes 2 and 3 are primarily responsible for clinically apparent cases.

### Age Distribution of Dengue Case-Patients

**Figure 3:**
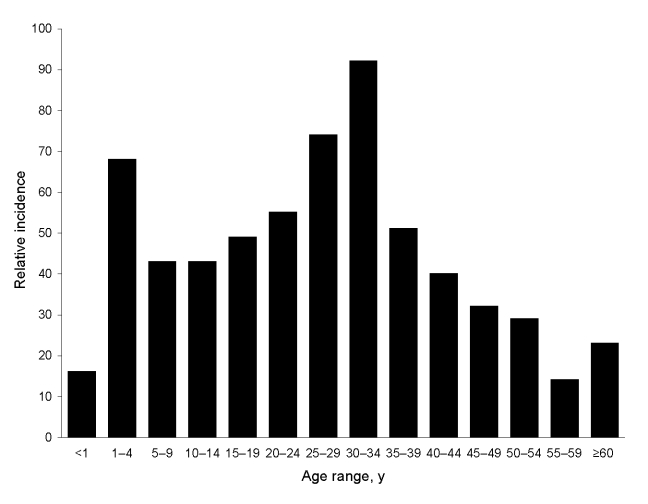
Clinically apparent dengue in different age groups in Sri Lanka, 2003–2006, Sri Lanka. Because true incidence data were not available, relative incidence of dengue infections by age cohort was estimated. We used Genetech data and known population of Colombo by age, to estimate relative incidence. The age group (>60 years) with the lowest transmission rate was used as a referent for calculating the fold difference between each remaining cohort and the referent.

When the age distribution of dengue-positive case-patients tested at Genetech during 2003–2006 was analyzed, we observed 2 peaks: the first in children <4 years of age and the second in adults 20–30 years of age (Figure 3). The peak of disease in adults was surprising because previous studies indicated that dengue transmission in Colombo was high and most adults were likely to be immune to infection (*8*). To further evaluate the peak of disease observed in young adults, we examined the age distribution of case-patients reported to the Ministry of Health during 1996–2006. These data showed a striking change in age distribution of dengue case-patients over this 11-year period (Technical Appendix). Before 2000, one large peak of cases was observed in children and few cases were observed for adults. After 2000, two peaks of reported disease were observed for children and young adults. Moreover, the mean age of reported DF/DHF cases has increased from 15 in 1996 to ≈25 in 2006.

### Phylogeny of DENVs

We reported that the DENV-3 strains isolated in Sri Lanka before and after the emergence of severe disease epidemics belonged to 2 distinct clades (*7*). To further characterize the DENVs responsible for recent epidemics in Sri Lanka, we isolated virus from serum samples collected in 2003 and 2004. The C6/36 mosquito cell line was inoculated by using serum samples from 220 samples that were positive by PCR for DENV. Virus was isolated from 181/220 specimens. The 181 isolates consisted of 18 DENV-1 strains, 76 DENV-2 strains, 64 DENV-3 strains, and 23 DENV-4 strains. To identify DENV genotypes that have been circulating in this country over the past 3 decades, we sequenced representative isolates from 2003 and 2004, as well as other isolates from Sri Lanka in our collection. All virus strains and sequences used for this analysis are listed in the Appendix Table. When grouping each DENV serotype into different genotypes, we relied on the groups and nomenclature described by Rico-Hesse for the 4 serotypes (*5*). Genotypes are named on the basis of country of origin of the earliest isolates and not necessarily on current distribution of viruses.

### Phylogeny of DENV-1 Strains Collected during 1983–2004

**Figure 4:**
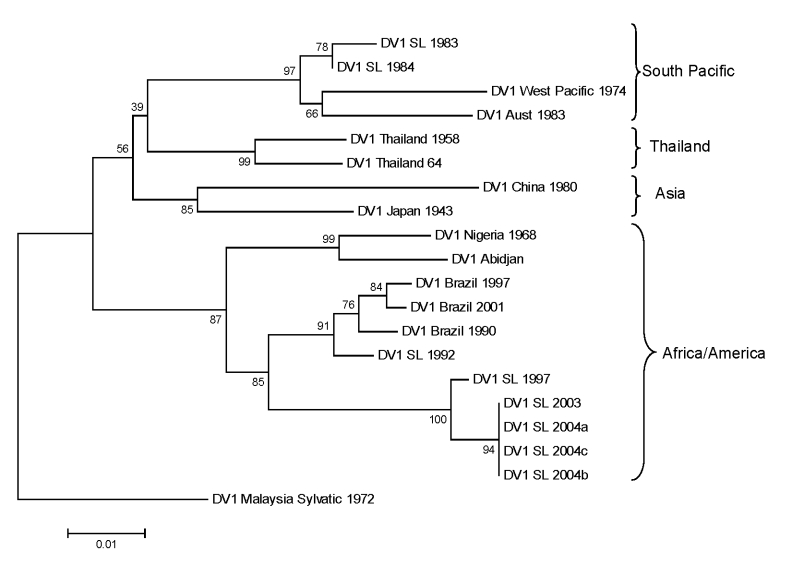
Phylogram of dengue serotype 1 viruses (DENV-1) from Sri Lanka (SL), 1983–2004, and other DENV-1 viruses. The tree is based on a 498-bp fragment for positions 2056–2554 coding portions of envelope protein and nonstructural protein 1. Evolutionary history was inferred by using minimum evolution method (*12*). Percentages of replicate trees in which the associated taxa clustered in the bootstrap test (1,000 replicates) are shown next to the branches (*13*). Phylogenetic analyses were conducted in MEGA4 (*14*). The tree was rooted by using a DENV-1 sylvatic strain. Classification and naming of different DENV-1 genotypes is based on the report by Rico-Hesse (*5*). Scale bar represents number of base substitutions per site.

DENV-1 has been subdivided into 4 genotypes designated South Pacific, Asia, Thailand, and Africa/America (*5*). We evaluated the position of DENV-1 isolates from Sri Lanka within this established phylogeny. These isolates used were obtained in 1983, 1984, 1997, 2003, and 2004. A 498-nt fragment from positions 2056 to 2554 (E/NS1 junction) was used to create a phylogenetic tree. Results demonstrate that the DENV-1 genotype circulating in Sri Lanka has changed over the study period. The 2 isolates from Sri Lanka obtained in 1983 and 1984 belonged to the South Pacific genotype (Figure 4). Sometime during 1984–1997, the Africa/America DENV-1 genotype became established on Sri Lanka and continued to circulate through 2004; the South Pacific genotype has not been detected during the past 8 years.

### Phylogeny of DENV-2 Strains Collected during 1981–2004

**Figure 5:**
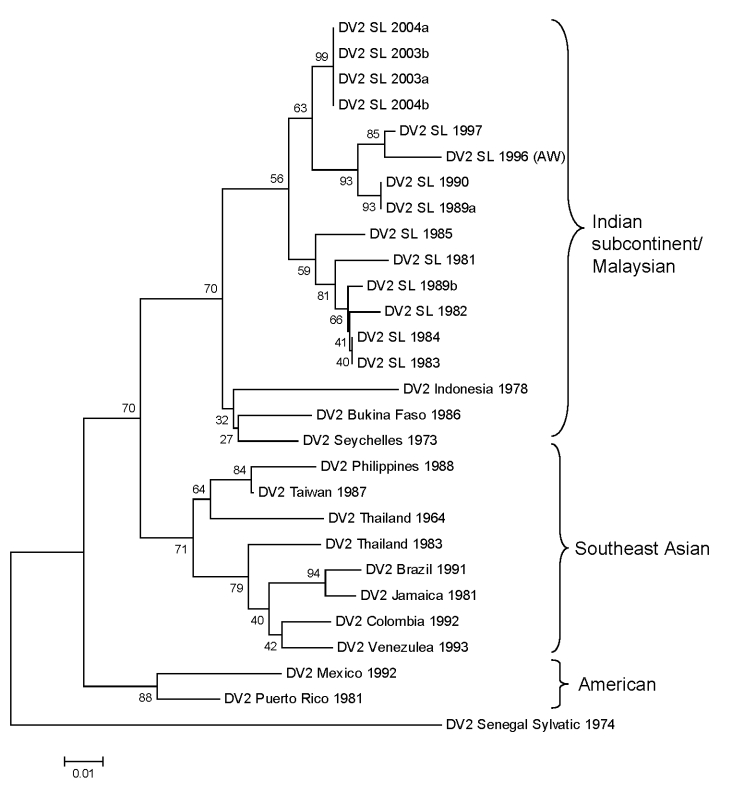
Phylogram of dengue serotype 2 viruses (DENV-2) from Sri Lanka (SL), 1981–2004, and other DENV-2 viruses. The tree is based on a 239-bp fragment for positions 2311–2550 coding for amino acids at the envelope protein/nonstructural protein 1 junction. The tree was constructed as described in Figure 4 and was rooted by using a DENV-2 sylvatic strain. Classification and naming of different DENV-2 genotypes is based on the report by Rico-Hesse (*5*). Scale bar represents number of base substitutions per site.

DENV-2 has been subdivided into 4 genotypes designated Malaysian/Indian subcontinent, Southeast Asian, American, and West African (Sylvatic) (*5*). The Sri Lankan DENV-2 strains in our collection were isolated in 1981, 1982, 1983, 1984, 1985, 1989, 1990, 1996, 1997, 2003, and 2004. We sequenced the 239-nt fragment from positions 2311–2550 (E/NS1 junction) and generated a phylogenetic tree by using this sequence and existing sequences in GenBank from representative DENV-2 strains. All DENV-2 isolates from Sri Lanka are closely related and belong to the Indian subcontinent/Malaysia genotype (Figure 5). Moreover, there is no evidence for the recent introduction of a DENV-2 strain from outside the island because the DENV-2 strains from Sri Lanka are more closely related to one another than to any other DENV-2 strain used in this analysis.

### Phylogeny of DENV-3 Strains Collected during 1983–2004

DENV-3 has been divided into 4 genotypes designated Southeast Asian/South Pacific (I), Thailand (II), Indian subcontinent (III), and American (IV) (*5*,*15*). Previous studies have demonstrated that all DENV-3 strains from Sri Lanka isolated in the 1980s and 1990s belong to Indian subcontinent genotype (III) (*7*,*15*). Within genotype III, DENV3 strains from Sri Lanka form 2 distinct clades linked to mild (IIIA) and severe (IIIB) disease epidemics on the island (*7*). DENV-3 IIIB viruses are most closely related to East African strains, which indicates that IIIB viruses linked to severe disease in Sri Lanka are likely to have been introduced from East Africa (*7*). In 1994, a DENV-3 genotype III from South Asia or East Africa was also introduced into Latin America where it is now well established and responsible for severe disease epidemics (*7*).

**Figure 6:**
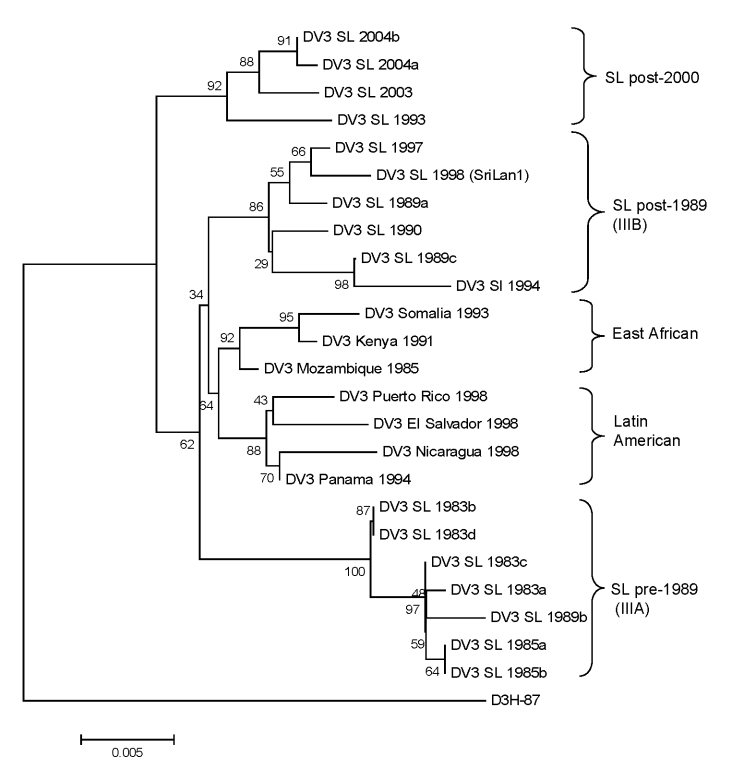
Phylogram of dengue serotype 3 (DENV-3) genotype III viruses from Sri Lanka (SL), 1981–2004, and other DENV-3 genotype III viruses. The tree is based on a 966-bp fragment for positions 179–1144 coding for a portion of the capsid protein, all of the premembrane protein, and a portion of the envelope protein. The tree was constructed as described in Figure 4 and rooted by using a DENV-3 genotype I virus (H87). Naming of the different groups within DENV-3 genotype III is based on the report by Messer et al. (*7*). Scale bar represents number of base substitutions per site

Considering the backdrop of the recent expansion of DENV-3 genotype III viruses, we were interested in determining the relationship of DENV-3 genotype III strains from Sri Lanka isolated in 2003 and 2004 to other DENV-3 genotype III viruses currently circulating in Africa, the Americas, and the Indian subcontinent. We sequenced the 966-nt fragment from positions 179–1144 (a portion of C, all of preM, and a portion of E) and created a tree by using our sequences and existing sequences in GenBank from representative DENV-3 genotype III strains. The DENV-3 sequences used were from isolates obtained in Sri Lanka in 1983, 1984, 1985, 1989, 1990, 1993, 1994, 1997, 1998, 2003, and 2004. As demonstrated previously by our group, DENV-3 genotype III consists of pre (IIIA)– and post (IIIB)–1989 clades from Sri Lanka, as well as Latin American and East African clades (Figure 6) (*7*). The DENV-3 strains from Sri Lanka isolated in 2003 and 2004 form a new, distinct clade that is closely related but distinct from the DENV-3 clade IIIB viruses that were isolated in the 1990s. This new 2003–2004 clade includes an isolate from 1993, which strongly suggests that the clade is derived from strains that have been on the island for some time.

### Phylogeny of DENV-4 Strains Collected during 1978–2004

**Figure 7:**
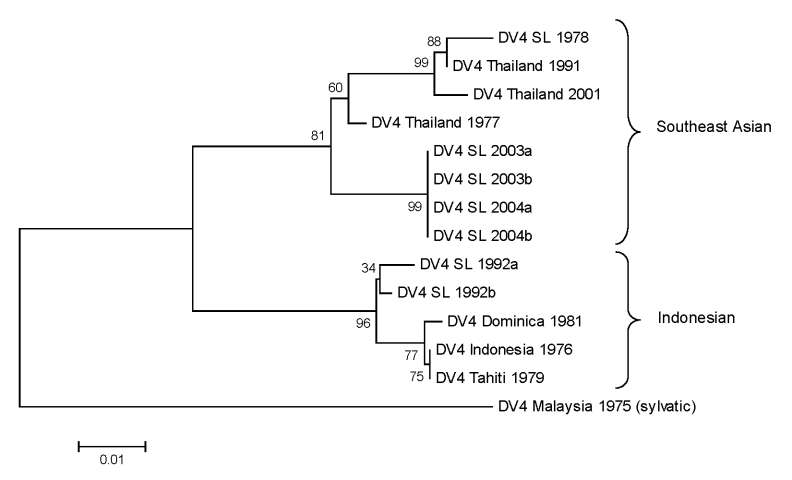
Phylogram of dengue serotype 4 viruses (DENV-4) from Sri Lanka (SL), 1978–2004, and other DENV-4 viruses. The tree is based on a 296-bp for positions 787–1083 coding for portions of premembrane and envelope proteins. The tree was constructed as described in Figure 4 and rooted by using a sylvatic DENV-4 strain. Classification and naming of different DENV-4 genotypes is based on the report by Rico-Hesse (5). Scale bar represents number of base substitutions per site.

The phylogeny of DENV-4 has not been studied as extensively as the other serotypes. This serotype can be broadly separated into 2 genotypes designated Southeast Asian (I) and Indonesian (II) (*5*). The Southeast Asian genotype strains are primarily from Asia, whereas the Indonesian group has a broad distribution in Asia and the Americas. A 296-nt fragment from positions 787–1083 (preM/E junction) was used to create a phylogenetic tree. DENV-4 strains from Sri Lanka isolated in 1978 and in 2003–2004 group with the Southeast Asian genotype, which indicates that this genotype is established on the island (Figure 7). Two DENV-4 isolates from 1992 belong to the Indonesian genotype and likely represent a transient introduction (Figure 7).

## Discussion

Although dengue has been a problem in the Indian subcontinent for at least the past 50 years, the disease and its viruses remain incompletely studied in the region (*16*–*18*). Over the past 2 decades, the epidemiology of dengue has changed and regular epidemics of DF and DHF have been reported in Sri Lanka, India, the Maldive Islands, Bangladesh, and Pakistan (*8*,*19*–*23*). We need a better understanding of the epidemiology of dengue in this region to develop and implement effective control programs and to most effectively use dengue vaccines that are currently in clinical trials.

We have investigated the emergence of DHF in Sri Lanka by analyzing samples sent for diagnostic testing to the Genetech Research Institute in Colombo. This institute received 3,833 samples for testing during 2003–2006. On an annual basis, the proportion of samples positive for dengue was 39% in 2003, 22% in 2004, 18% in 2005, and 32% in 2006. The low proportion of positive samples in 2004 and 2005 compared with other years was unexpected because one would expect a greater proportion of positive cases during an epidemic year such as 2004. During the 2004 epidemic, there was widespread fear of dengue, and indiscriminate testing of fever cases is likely to have led to the overall lower proportion of positive cases.

We have demonstrated that all 4 serotypes cocirculate in Sri Lanka and were responsible for clinically apparent cases detected during 2003–2006. DENV types 2 and 3 were responsible for most human cases; types 1 and 4 were relatively rare. DENV types 2 and 3 native to the region may be more pathogenic than the other serotypes and thus may be recovered more frequently during human surveillance.

Viruses isolated during 1978–2004 were sequenced to understand the origin and evolution of DENV in Sri Lanka. For DENV-1, isolates obtained in Sri Lanka in the 1980s belong to the South Pacific genotype, whereas more recent isolates belong to the American/African genotype. These results indicate that the South Pacific genotype of DENV-1 was replaced after a new introduction of the American/African genotype of DENV-1. All DENV-2 isolates from Sri Lanka belong to a single (Indian subcontinent/ Malaysian) genotype. There is no evidence for introduction of DENV-2 from other areas because DENV-2 strains from Sri Lanka were more related to one another than to any other strain used in this analysis. All DENV-3 strains from Sri Lanka belonged to genotype III. However, in 1989 and again in 2000, the dominant clade of DENV-3 genotype III was replaced by a new clade of genotype III (*7*). In 1989, the lineage replacement was most likely caused by the introduction of DENV-3 from outside Sri Lanka. In 2000, the dominant lineage of DENV-3 was replaced by a previously rare lineage from Sri Lanka. The oldest (1978) and most recent isolates of DENV-4 belong to the Southeast Asian genotype, which indicates that this genotype is established on the island.

Perhaps the most striking feature of the epidemiology of dengue in Sri Lanka is the abrupt, stepwise increase in the number of severe disease cases in 1989 and again in 2000. In previous studies, we have highlighted the potential role of a shift in the circulating clade of DENV-3 from genotype IIIA to IIIB in this sudden emergence of severe disease in 1989 (*7*). Recent studies have also demonstrated that DENV-3 clade IIIB viruses replicate and disseminate better in the vector than clade IIIA viruses (*24*). This finding may explain the explosive spread of IIIB and closely related viruses within the region as well as into Latin America. The stepwise increase in cases after 2000 was accompanied by appearance of another clade of DENV-3 genotype III viruses that have replaced the clade IIIB viruses. Thus, evolution within DENV-3 genotype III continues to be linked to changes in disease severity in Sri Lanka. A similar phenomenon has also been reported by Bennett et al. (*25**,**26*). They compared data for 20 years from Puerto Rico for DENV-2 and DENV-4 and observed replacement of dominant clades by a previously rare lineage in the population or by viruses introduced from outside Puerto Rico. Clade replacement was linked to positive selection in the NS2A gene for DENV-4 and the E gene for DENV-2. Further studies are needed to assess if mutations in specific genes are also linked to emergence of new clades of DENV-3 in Sri Lanka.

When analyzing data collected at Genetech during 2003–2006, we observed a peak of dengue in young adults (20–30 years of age). Analysis of national data collected during 1996–2005 demonstrated that the disease peak in adults is a recent phenomenon and occurred after 2000. In areas with high transmission, where the virus has been historically established, most adults are likely to be immune because of childhood infections. The changing age structure may be indicative of the virus moving into new areas with many susceptible adults. During the large epidemics that occurred after 2000, many cases were reported from regions of the country where few cases have been reported (Epidemiology Unit, Ministry of Health, unpub. data). To better understand the molecular epidemiology and changing age distribution of dengue in Sri Lanka, laboratory-supported, population-based, active surveillance studies are needed.

## Supplementary Material

Appendix Table DENV sequences used to construct phylogenetic trees*

Technical Appendix Severe Dengue Epidemics in Sri Lanka, 2003-2006
