# Erratum—Vol. 15, No. 2

**DOI:** 10.3201/eid1504.080926

**Published:** 2009-04

The name of Vasanthi Thevanesam should have been included in the author list for the article Severe Dengue Epidemics in Sri Lanka, 2003–2006 (N. Kanakaratne et al.). Prof Thevanesam is affiliated with the University of Peradeniya Faculty of Medicine, Peradeniya, Sri Lanka. The article has been corrected online (www.cdc.gov/eid/content/15/2/192.htm).

