# Supplementary material for: Severe Dengue Epidemics in Sri Lanka, 2003–2006
Source: Emerg Infect Dis. 2009 Feb;15(2):192–9. doi: 10.3201/eid1502.080926 (PMC2662655; doi:10.3201/eid1502.080926)
Supplement: Appendix Table — DENV sequences used to construct phylogenetic trees* [file 08-0926_appT-s1.pdf]

Appendix Table. DENV sequences used to construct phylogenetic trees*

Virus	Taxon name	Source of virus or sequence	GenBank accession no.
DENV-1	DV1 West Pacific 1974	GenBank	U88535
	DV1 Aust 1983	GenBank	AB074761
	DV1 Japan 1943	GenBank	AB074760
	DV1 1980	GenBank	AF350498
	DV1 Thailand 1964	GenBank	AF180818
	DV1 Thailand 1958	GenBank	D10513
	DV1 Abidjan	GenBank	AF298807
	DV1 Nigeria 1968	GenBank	AF425625
	DV1 Brazil 2001	GenBank	AF513110
	DV1 Brazil 1997	GenBank	AF311956
	DV1 Brazil 1990	GenBank	AF226685
	DV1 Malaysia (Sylvatic) 1972	GenBank	EF457905
	DV1 SL 1983	D. Gubler, CDC	FJ225443
	DV1 SL 1984	D. Gubler, CDC	FJ225444
	DV1 SL 1992	D. Gubler, CDC	FJ225445
	DV1 SL 1997	A.M. de Silva, UNC	FJ225446
	DV1 SL 2003a	A.M. de Silva, UNC	FJ225447
	DV1 SL 2004a	A.M. de Silva, UNC	FJ225448
	DV1 SL 2004b	A.M. de Silva, UNC	FJ225449
	DV1 SL 2004c	A.M. de Silva, UNC	FJ225450
DENV-2	DV2 Senegal (Sylvatic) 1974	GenBank	M32957
	DV2 Mexico 1992	GenBank	U91888
	DV2 Venezuela 1993	GenBank	U91870
	DV2 Colombia 1992	GenBank	U91876
	DV2 Jamaica 1981	GenBank	M32950
	DV2 Brazil 1991	GenBank	U91867
	DV2 Thailand 1983	GenBank	M32947
	DV2 Thailand 1964	GenBank	M32941
	DV2 Taiwan 1987	GenBank	M32949
	DV2 Philippines 1988	GenBank	M32932
	DV2 Seychelles 1973	GenBank	M32952
	DV2 Bukina Faso 1982	GenBank	M32956
	DV2 Indonesia 1978	GenBank	M32934
	DV2 SL 1981	GenBank	M32938
	DV2 SL 1982	GenBank	M32940
	DV2 SL 1983	D. Gubler, CDC	FJ225451
	DV2 SL 1984	D. Gubler, CDC	FJ225452
	DV2 SL 1985	GenBank	M32953
	DV2 SL 1989a	D. Gubler, CDC	FJ225453
	DV2 SL 1989b	D. Gubler, CDC	FJ225454
	DV2 SL 1990	D. Gubler, CDC	FJ225455
	DV2 SL 1996	A.M. de Silva, UNC	FJ225456
	DV2 SL 1997	A.M. de Silva, UNC	FJ225457
	DV2 SL 2003a	A.M. de Silva, UNC	FJ225458
	DV2 SL 2003b	A.M. de Silva, UNC	FJ225459
	DV2 SL 2004a	A.M. de Silva, UNC	FJ225460
	DV2 SL 2004b	A.M. de Silva, UNC	FJ225461
DENV-3	DV3 SL 2003a	A.M. de Silva, UNC	FJ225463
	DV3 SL 2004a	A.M. de Silva, UNC	FJ225464
	DV3 SL 2004b	A.M. de Silva, UNC	FJ225465
	DV3 SL 1983a	GenBank	AF547225
	DV3 SL 1983b	GenBank	AF547227
	DV3 SL 1983c	GenBank	AF547228
	DV3 SL 1983d	GenBank	AF547229
	DV3 Mozambique 1985	GenBank	AF547237
	DV3 SL 1985a	GenBank	AF547241
	DV3 SL 1985b	D. Gubler, CDC	FJ225462
	DV3 SL 1989a	GenBank	AF547230
	DV3 SL 1989b	GenBank	AF547231
	DV3 SL 1989c	GenBank	AF547232
	DV3 SL 1990	GenBank	AF547233
	DV3 Kenya 1991	GenBank	AF547239
	DV3 Somalia 1993	GenBank	AF547240
	DV3 SL 1993	GenBank	AF547234
	DV3 Panama 1994	GenBank	AF547247
	DV3 Sl 1994	GenBank	AF547235

	DV3 SL 1997	GenBank	AF547242
	DV3 El Salvador 1998	GenBank	AF547259
	DV3 Nicaragua 1998	GenBank	AF547245
	DV3 Puerto Rico 1998	GenBank	AF547258
	DV3 SL 1998	GenBank	AF547243
	D3H-87	GenBank	M93130
DENV-4	DV4 Dominica 1981	GenBank	AY152360
	DV4 Thailand 1991	GenBank	AY618990
	DV4 Thailand 2001	GenBank	AY618992
	DV4 Thailand 1977	GenBank	AY618991
	DV4 Malaysia (Sylvatic) 1975	GenBank	EF457906
	DV4 China	GenBank	AF289029
	DV4 Indonesia 1976	GenBank	U18429
	DV4 Tahiti 1979	GenBank	U18438
	DV4 Sri Lanka 1978	GenBank	AY550909
	DV4 SL 1992a	D. Gubler, CDC	FJ225466
	DV4 SL 1992b	D. Gubler, CDC	FJ225467
	DV4 SL 2003a	A.M. de Silva, UNC	FJ225468
	DV4 SL 2003b	A.M. de Silva, UNC	FJ225469
	DV4 SL 2004a	A.M. de Silva, UNC	FJ225470
	DV4 SL 2004b	A.M. de Silva, UNC	FJ225471

*DENV, dengue virus; CDC, Centers for Disease Control and Prevention (Fort Collins, CO, USA); UNC, University of North Carolina School of Medicine (Chapel Hill, NC, USA); SL, Sri Lanka.
