# Supplementary material for: Severe Dengue Epidemics in Sri Lanka, 2003–2006
Source: Emerg Infect Dis. 2009 Feb;15(2):192–9. doi: 10.3201/eid1502.080926 (PMC2662655; doi:10.3201/eid1502.080926)

Severe Dengue Epidemics in Sri Lanka, 2003–2006

Technical Appendix

Age distribution of dengue case-patients reported to the Epidemiology Unit, Ministry of Health (annual data during 1996–2006). Note that the age distribution of case-patients shifts from a single peak in children only before 2000 to 2 peaks, 1 in children and 1 in young adults, after 2000.

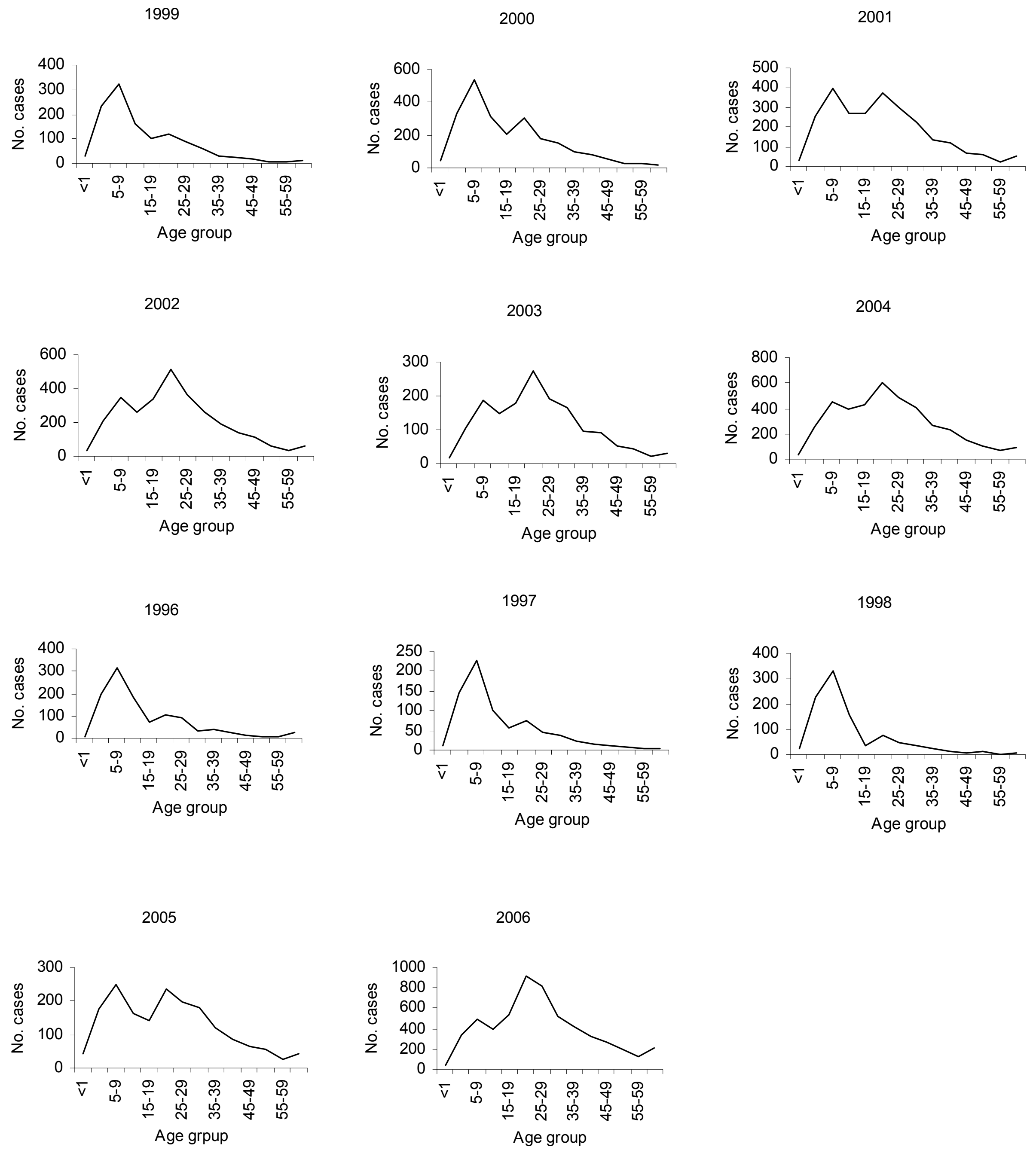

Supplement: Technical Appendix — Severe Dengue Epidemics in Sri Lanka, 2003-2006 [file 08-0926_Techapp-s2.pdf]
